# R-split-EPOCH plus high dose methotrexate in untreated diffuse large B cell lymphoma with *MYC* rearrangement or double expression of *MYC* and *BCL-2*

**DOI:** 10.7150/jca.52958

**Published:** 2021-02-02

**Authors:** Peng Sun, Yu Wang, Cui Chen, Hang Yang, Man Nie, Xiao-Qing Sun, Xiao-Hua He, Wen-Qi Jiang, Zhi-Ming Li

**Affiliations:** 1 Department of Medical Oncology, Sun Yat-Sen University Cancer Center, 651 Dong Feng RD East, Guangzhou, Guangdong Province 510060, P. R. China.; 2 State Key Laboratory of Oncology in South China, Collaborative Innovation Center for Cancer Medicine, 651 Dong Feng RD East, Guangzhou, Guangdong Province 510060, P. R. China.; 3 Department of Oncology, the First Affiliated Hospital, Sun Yat-Sen University, 58 Zhongshan Road Ⅱ, Guangzhou, Guangdong Province 510080, P. R. China.

**Keywords:** R-split-EPOCH, high dose methotrexate (HD-MTX), diffuse large B cell lymphoma (DLBCL), *MYC*, double expression

## Abstract

**Purpose:** Diffuse large B cell lymphoma (DLBCL) with *MYC* rearrangement or double expression of *MYC* and BCL-2 (DE DLBCL) has a relatively poor prognosis and does not respond well to standard R-CHOP. In the current study, we aimed to investigate the efficacy and safety of R-split-EPOCH plus high dose methotrexate (HD-MTX) in the particular patient population.

**Methods:** A total of 28 patients diagnosed with DE DLBCL or DLBCL with *MYC* rearrangement between January 2015 and December 2018 were included and retrospectively analyzed. All the participants underwent R-split-EPOCH plus HD-MTX as introduction therapy, with split infusion of etoposide, doxorubicin, and vincristine for 48 hours on D1-2 and D10-11, respectively.

**Results:** The overall objective response (ORR) rate was 100%, with 24 (85.7%) complete response (CR) and 4 (14.3%) partial response (PR). The CR rate was 76.9% and 93.3% for DLBCL patients with *MYC* rearrangement and DE DLBCL patients, respectively. The 1- and 3-year PFS rate was 100% and 74.9%, respectively. The 1- and 3-year OS rate was 100% and 92.9%, respectively. Grade 3/4 non-hematological toxicity and grade 3/4 hematological toxicity occurred in 50% and 85.7% of patients, respectively. No treatment-related death was reported.

**Conclusions:** R-split-EPOCH plus HD-MTX regimen is an effective and feasible treatment option for DE DLBCL and DLBCL with *MYC* rearrangement.

## Introduction

Diffuse large B cell lymphoma (DLBCL) is the most frequent type of non-Hodgkin's lymphomas, accounting for 30-40% of newly diagnosed lymphoma cases in China 1. The combination of rituximab plus CHOP (cyclophosphamide, doxorubicin, vincristine, prednisone; R-CHOP) has been established as the standard frontline care for DLBCL by a series of prospective trials, and has greatly improved the clinical outcome of DLBCL in the past two decades 2-4.

However, DLBCL is molecularly heterogeneous and appropriately 30% of DLBCL patients would ultimately develop refractory or relapse disease after receiving first-line treatment of R-CHOP 3. *MYC* 8q24 rearrangement is a relatively frequent molecular event in DLBCL and is found to be strongly associated with poor prognosis of DLBCL treated with R-CHOP 5, 6. Recently, DLBCL patients harboring genetic rearrangement of* MYC*, with concurrent translocation of *BCL2* and/or *BCL6*, which are found to have significant shorter survival than patients without genetic alterations of *MYC,* have been recognized as a novel category of high-grade B cell lymphoma (HGBCL) by the revised 4^th^ edition of World Health Organization classification 7. In addition, DLBCL patients with co-expression of *MYC* (≥40%) and *BCL2* (≥50%) are now considered as double expressor lymphomas (DE DLBCL) 8. Although DE DLBCLs don't form an independent entity in the revised World Health Organization classification, they serve as biomarkers for unfavorable prognosis in the R-CHOP era 8.

Given that R-CHOP doesn't show sufficient efficacy in DLBCL with *MYC* rearrangement and DE DLBL, the use of intensive regimens has been prompted in these patients. The infusional dose-adjusted etoposide, prednisone, vincristine, cyclophosphamide, and doxorubicin (DA-EPOCH) combination is a dose-intense immunochemotherapy and shows promising results in Burkitt's lymphoma, which is recognized a highly proliferative lymphoma with *MYC*-rearrangement. In untreated large B cell lymphoma, DA-EPOCH showed a high complete response rate of 92% and promising data of PFS and OS 9. Furthermore, rituximab plus DA-EPOCH (DA-EPOCH-R) resulted in a 1-year PFS rate of 85% 10, 11. In 2018, Dodero et al. compared the outcome of de novo DE DLBCL patients treated with DA-EPOCH-R and R-CHOP, demonstrating that DA-EPOCH-R could achieve a better PFS for young DE DLBCL patients 12. In patients with *MYC*-rearranged aggressive B-cell lymphomas, the currently available data suggests that DA-EPOCH-R has a relatively higher response rate and in some instances with improved survival 13.

Despite the lack of prospective data regarding the DA-EPOCH-R in DLBCL with *MYC* rearrangement and DE DLBCL patients, the regimen has been widely accepted as an option for these patients. However, DA-EPOCH-R was more toxic in previous studies and less tolerable in elderly and fragile patients. Splitting the administration of chemotherapy agents is a rational option without compromising the dose-intensity, and has been succeeded in a series of solid cancers and hematological malignancies 14, 15. Kreher et al. introduced R-split-CHOP to treat elderly patients with DLBCL, suggesting that R-split-CHOP could be safely used in elderly patients who were at risk of treatment-related complications 15. In our institution, we have used R-split-EPOCH plus high dose methotrexate (HD-MTX) to treat DLBCL with *MYC* rearrangement and DE DLBCL since the year of 2015. In the current study, we aimed to investigate the efficacy and safety of R-split-EPOCH plus HD-MTX in the particular patient population.

## Materials and methods

### Ethics approval and consent to participate

All patients signed consent for the collection and procession of clinic-pathological data throughout the period covered by this study. The study was approved by the Bioethics Committee of our institution for a retrospective analysis of the collected data and undertaken in accordance with the ethical standards of the World Medical Association's Declaration of Helsinki. We have uploaded the essential raw data onto the Research Data Deposit (RDD) public platform (https://www.researchdata.org.cn).

### Patients

All patients diagnosed with DLBCL between January 2015 and December 2018 were retrospectively reviewed in our institution. Included patients met the following criteria: (a) the disease was pathologically diagnosed as DLBCL with *MYC* rearrangement or with co-expression of *MYC* (≥40%) and *BCL2* (≥50%); (b) complete clinical and treatment information were available; (c) the patients were between 18 and 80 years of age; (d) no involvement of CNS; (e) no antitumor treatment was given before admission; and (f) the patient presented with at least one measurable lesion. The exclusion criteria were (a) patients with other types of malignancy and (b) patients with insufficient renal function or hepatic function.

### Treatment protocol

Rituximab (375 mg/m^2^) was given before the start of EPOCH. Etoposide 50 mg/m² per day, doxorubicin 10 mg/m² per day, and vincristine 0.4 mg/m² per day were all infused for 48 hours on D 1-2 and D 10-11, while cyclophosphamide 375 mg/m² was intravenously administered on D3 and D12. Prednisone was orally administered as 60 mg/m² a day on D1-3 and D10-11. MTX (3 g/m^2^) was infused for 12 hours on D 12, followed 12 hours later by leucovorin 30 mg every 6 hours. MTX levels were measured every 12 hours until the MTX level was <1×10^-7^ mol/L. The chemotherapy treatment was repeated every 3 weeks, and total cycles of chemotherapy were decided by physician. Dose escalation was not allowed in this study. Granulocyte colony-stimulating factor (G-CSF) was applied at the physician's discretion and prophylactic G-CSF was not routinely used. CNS prophylaxis with intrathecal methotrexate and cytarabine was administered to all patients but those with lumbar diseases. The therapy was ceased if the disease progressed or intolerable toxicity occurred. According to the physicians' decisions and patients' willingness, patients underwent consolidation therapy [autologous stem-cell transplantation (ASCT) or radiation].

### Treatment evaluation and toxicity

Pretreatment evaluations included physical examination, bone marrow aspiration and biopsy, routine laboratory tests, electrocardiogram, echocardiogram. ^18^F-fluorodeoxyglucose (^18^F-FDG) PET/CT scan was selected for disease staging at baseline in all patients. Radiological scan was performed post cycle 2, cycle 4 and cycle 6, and all patients underwent interim ^18^F-FDG PET/CT scan. Response was assessed by investigators per Revised Response Criteria for Malignant Lymphoma 16. After completing treatment, the patients were evaluated by repeat radiological scans every 3 months for the first two years and then every 6 months for years 3-5. Upon cessation of treatment, each patient was followed up every 3 months at the clinic or by telephone interview until 5 years. Treatment-related adverse events were evaluated with the Common Terminology Criteria for Adverse Events (CTCAE) version 4.0 17.

### Statistical analyses

The treatment responses of different groups were compared using the chi-square or Fisher's exact tests. OS was calculated as the time from diagnosis to the date of death or last follow-up visit, and PFS was calculated as the time from diagnosis to relapse, progression, death or the date of the last follow-up visit. Survival curves were obtained by the Kaplan-Meier method. The statistical analyses were performed using the Statistical Package for the Social Sciences version 22.0.

## Results

### Patient characteristics

Finally, our study enrolled a total of 28 patients. The 28 participants had a median age of 47.5 years (range 27-69), 15 (53.6%) were male, and 20 (71.4%) had Ann Arbor stage III and IV disease. According to international prognostic index (IPI), 12 (42.9%) patients had high-intermediate or high-risk disease. Fifteen patients were diagnosed as DE DLBCL. The rest 13 patients all had *MYC* rearrangement, and among them 4 double-hit lymphomas (DHL) and 5 triple-hit lymphomas (THL) were identified. All the patients' characteristics were summarized in Table 1.

### Efficacy and survival

The median number of chemotherapy cycles was 6 (range: 2-8). All patients enrolled underwent at least two cycles of chemotherapy and were thus could be evaluated for efficacy. Three patients switched to R-CHOP treatment for intolerable toxicity, and three patients didn't receive HD-MTX after four cycles of chemotherapy. Meanwhile, a total of 24 patients received CNS prophylaxis with intrathecal methotrexate and cytarabine. Two patients with complete response (CR) and one patient with partial response (PR) underwent radiotherapy after chemotherapy. Only one patient who had a CR underwent ASCT after initial chemotherapy.

**Figure 1:**
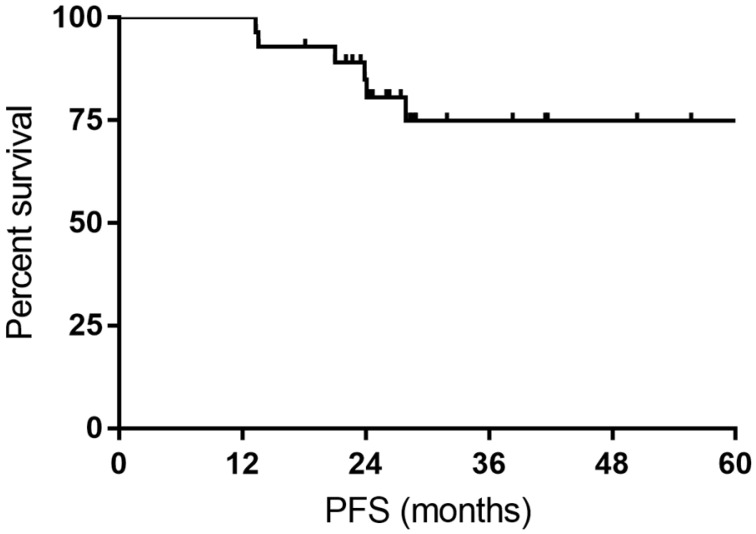
Kaplan-Meier curves for progression-free survival (PFS).

**Figure 2:**
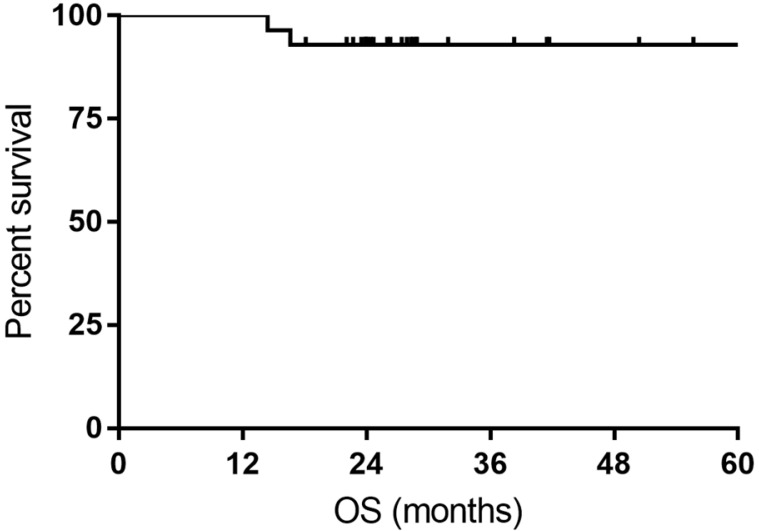
Kaplan-Meier curves for overall survival (OS).

All the responses were confirmed by ^18^FDG-PET/CT scan. The overall objective responses rate was 100%, with 24 (85.7%) CR and 4 (14.3%) PR. The CR rate was 76.9% and 93.3% for DLBCL patients with *MYC* rearrangement and DE DLBCL patients, respectively (Table 2). With a median follow-up time of 27.7 months (range: 14.4-63.4months), the median OS time and PFS time were neither reached. Six patients experienced relapse of lymphoma, and two patients died of progression of THL. The 1- and 3-year PFS rate was 100% and 74.9%, respectively. The 1- and 3-year OS rate was 100% and 92.9%, respectively (Figure 1 and Figure 2).

### Toxicity

All patients had available toxicity data for safety analysis. No treatment-related death was reported. Table 3 lists the detailed toxicity data. Generally, hematological toxicity was more common than non-hematological toxicity in our study. All patients received G-CSF for one or more cycles of therapy. Grade 3/4 neutropenia, anemia and thrombocytopenia occurred in 85.7%, 57.1%, and 39.3% of patients, respectively. 16 (57.1%) patients developed febrile neutropenia and 7 (25%) patients developed pneumonia during the therapy. The most frequent grade 3/4 non-hematological toxicity was mucositis (39.3%). Of note, concentration of MTX beyond permissible standard was reported in two patients (7.1%) at 48 hours after infusion, which discontinued the HD-MTX in subsequent cycles.

## Discussion

To the best of our knowledge, our study is the first one observing the preliminary efficacy and safety profile of R-slit-EPOCH plus HD-MTX in untreated DLBCL with *MYC* rearrangement or double expression of *MYC* and *BCL-2.* In the current study, the regimen of R-slit-EPOCH plus HD-MTX gave a relatively high response rate and yielded promising survival. Meanwhile, the regimen was also well tolerated by most of patients and showed reliable safety.

DE DLBCL is recognized as a high-risk subtype of DLBCL and has a poor clinical outcome 8, 18. Several colleagues attempted to treat this particular disease with intensive chemotherapeutic regimens, of which DA-EPCOH-R was an optional selection. Dodero et al. 12 adopted DA-EPOCH-R for DE DLBCL patients and compared the survival in DA-EPOCH-R and R-CHOP cohorts. The data demonstrated a better 2-year OS of DA-EPOCH-R (90% vs 67% of R-CHOP, *p*= 0.07), whereas in patients younger than 65 years DA-EPOCH-R obtained significantly better 2-year PFS and OS than R-CHOP. Although splitting the delivery of standard dose of R-EPOCH, our study showed inspiring response rate (ORR 100%, CRR 93.3%) in 15 DE DLBCL patients, with a 2-year PFS of 79.1% and a 2-year OS of 100%.

DLBCL with *MYC* rearrangement with or without *BCL2/BLC6* rearrangement is considered as extremely high-risk subtype of non-Hodgkin's lymphoma 16, 19. Due to the rarity, there is a lack of prospective studies guiding the treatment of these patients, and the current literature consists of retrospective data 20-23. To date, intensive regimens with higher response rates, such as DA-EOPCH-R and R-CODOX-M/R-IVAC, are now recommended for the frontline treatment of these patients. In a multicentre study, Dunleavy et al. evaluated the outcome of DA-EPOCH-R in aggressive B-cell lymphoma patients with *MYC* rearrangement, of which 24 participants were identified as DHL/THL 24. DA-EPOCH-R yielded a ORR of 87% in the whole cohort and a 4-year OS of 82%, as reported by Dunleavy et al. 24, revealing that DA-EPOCH-R obviated the adverse effect of *MYC* rearrangement and was a feasible treatment option for these patients. In another study from American, DA-EPOCH-R showed a high CRR of 68% and improved event free-survival and OS when compared with R-CHOP in DHL patients 22. Our study showed a comparable CRR of 76.9% and an encouraging 2-year OS of 84.6%, indicating R-split-EPOCH was also highly active in treating DLBCL with *MYC* rearrangement.

The major concern for DA-EPOCH-R in clinical practice is its relatively higher incidence of severe toxicities when compared with R-CHOP. In the prospective study by Dunleavy et al. 24, 63% of patients developed grade 3/4 neutropenia and 19% of patients experienced febrile neutropenia in the course of DA-EPOCH-R. Alliance/CALGB 50303 was an intergroup, randomized phase III study, which aimed to compare the efficacy of R-CHOP to DA-EPOCH-R in patients with untreated DLBCL 25. It prospectively evaluated the toxicity of DA-EPOCH-R in a large size (n=237), and significantly greater toxicity was found in DA-EPOCH-R cohorts compared with R-CHOP 25. Grade 3/4 hematological toxicities were reported in 97.5% of patients (73.3% in R-CHOP), while grade 3/4 non-hematological toxicities were reported in 72.2% of patients (42.2% in R-CHOP) 25. It was worth noting that 35.5% of patients occurred febrile neutropenia (17.7% in R-CHOP) when treating with DA-EPOCH-R 25. In order to reduce treatment toxicity, the 96-hour infusion of EPOCH was split up into two doses of 48-hour infusion in our study. As expected, our data showed lower incidence of grade 3/4 non-hematological toxicity (50%) and grade 3/4 hematological toxicity (85.7%) compared to Alliance/CALGB 50303. However, febrile neutropenia still occurred in 16 (57.1%) patients. Unlike the previous studies of DA-EPOCH-R, primary prophylactic use of granulocyte colony-stimulating factor (G-CSF) was not routinely prescribed to all patients. Most of grade 3/4 hematological toxicity events and febrile neutropenia developed after the first cycle of chemotherapy. When G-CSF was prophylactically used, hematological and febrile neutropenia could be well handled. Furthermore, no treatment-related death was observed. Generally speaking, most of patients could well tolerate and complete the therapy regimen in our study.

Central nervous system (CNS) involvement should always be paid attention to high-risk DLBCL. CNS involvement could occur at initial diagnosis or at the time of disease relapse. Previous data showed that the incidence of CNS involvement was 9.7% in DE DLBCL 18 and 13% in DHL 21, 23. Therefore, prophylaxis for CNS disease was important and necessary for these patients. In contrast to intrathecal injection of MTX or cytarabine, intravenous HD-MTX could penetrate the brain-blood barrier and was proved to be more effective in brain parenchyma. However, it is very difficult to delivery intravenous MTX with DA-EPOCH-R. We gave HD-MTX to patients on D12 following the second section of split-EPOCH. Of note, only two MTX-intoxication events were found and were ultimately relived with adequate detoxication. During the follow-up time, no CNS involvement was found. Our results showed that the addition of HD-MTX to R-split-EPOCH was feasible and effective in prophylaxis of CNS.

There are also several limitations in our study. This single center study inevitably has patient selection bias due to its retrospective nature. On the other hand, the study is of insufficient size for the exploratory analyses of prognostic factors. Nevertheless, we are still encouraged by these preliminary efficacy and survival data in this study. All patients underwent ^18^FDG-PET/CT scan to confirm treatment response, and these findings strongly indicate that our regimen is highly active in DE DLBCL or DLBCL with *MYC* rearrangement. We are now planning to conduct a prospective study to further validate our findings.

In summary, R-split-EPOCH plus HD-MTX regimen as first-line treatment demonstrated high response rates and favorable survival in DE DLBCL and DLBCL with *MYC* rearrangement. Meanwhile, treatment-related toxicities were well tolerated and acceptable in this study. Our results provide some preliminary data to support an optional and safe treatment for these patients.

## Figures and Tables

**Table 1:** Baseline clinical characteristics of 28 patients

	Number	%
**Age (years)**		
Mean	48.2	
Median (Range)	47.5 (27-69)	
≤60	23	82.1
>60	5	17.9
**Gender**		
Male	15	53.6
Female	13	46.4
**ECOG score**		
0-1	26	92.9
≥2	2	7.1
Raised LDH	12	42.9
Stage 3-4	20	71.4
**IPI**		
0-1	10	35.8
2	6	21.4
3	9	32.1
4-5	3	10.7
BM involvement	4	14.3
> 1 extranodal sites	17	60.7
Double hit	4	14.3
Triple hit	5	17.9
*MYC* rearrangement	13	46.4
Double expressor	15	53.6

ECOG, eastern cooperative oncology group; LDH, lactate dehydrogenase; IPI, international prognostic index; BM, bone marrow.

**Table 2:** Evaluation of treatment response

Response	Overall	DE DLBCL	DLBCL with *MYC* rearrangement	*P* value
CR (%)	24 (85.7)	14 (93.3)	10 (76.9)	0.311
PR (%)	4 (14.3)	1 (6.7)	3 (23.1)	

CR, complete remission; PR, partial remission; DE DLBCL, diffuse large B cell lymphoma with double expression of *MYC* and *BCL2.*

**Table 3:** Toxicities

	All events (%)	Grade 1 (%)	Grade 2 (%)	Grade 3 (%)	Grade 4 (%)
**Hematologic toxicities**					
Neutropenia	28 (100)	1 (3.6)	3 (10.7)	4 (14.3)	20 (71.4)
Anemia	28 (100)	0	12 (42.9)	15 (53.6)	1 (3.6)
Thrombocytopenia	21 (75.0)	3 (10.7)	7 (25.0)	4 (14.3)	7 (25.0)
**Infectious complications**					
Febrile neutropenia	16 (57.1)	—	—	—	—
Pneumonia	7 (25.0)	—	—	—	—
Nausea/vomiting	28 (100)	5 (17.9)	17 (60.7)	6 (21.4)	0
Mucositis	28 (100)	3 (10.7)	14 (50.0)	11 (39.3)	0
Hepatotoxicity	22 (78.6)	16 (57.1)	4 (14.3)	2 (7.1)	0
Nephrotoxicity	8 (28.6)	6 (21.4)	1 (3.6)	1 (3.6)	0
Cardiotoxicity	8 (28.6)	7 (25.0)	1 (3.6)	0	0
Concentration of MTX beyond permissible standard	2 (7.1)	—	—	—	—

HD-MTX, high dose methotrexate.
